# Exploring the psychosocial characteristics of women with gambling disorder through a qualitative study

**DOI:** 10.3389/fpsyg.2023.1294149

**Published:** 2023-12-20

**Authors:** Ana Estévez, Laura Macía, Andrea Ontalvilla, Maite Aurrekoetxea

**Affiliations:** ^1^Psychology Department, University of Deusto, Bilbao, Spain; ^2^Department of Social and Human Sciences, University of Deusto, Bilbao, Spain

**Keywords:** gambling, women, gender perspective, social stigmatization, qualitative

## Abstract

**Introduction:**

Gambling disorder is a behavioral addiction that has been primarily male, but in the last few years, the age of onset has been equated between the sexes. The profile of female gamblers could be different from that of men. Consequently, this study analyzes the testimonies of women with gambling disorder to determine their specific characteristics (gambling motives, gambling preferences, and associated pathologies).

**Method:**

The sample comprises 18 women with gambling disorder aged between 30 and 68. Three discussion groups were held, and a “blind” inductive process was carried out to extract categories. The Atlas.Ti 22.0 program was used to recode and analyze the data.

**Results:**

Results show that women may start gambling due to abuse suffered in childhood, and often family members initiate women into gambling. Likewise, the onset of gambling could be a maladaptive way to manage negative emotions. Also highlighted is the predominance of feelings of social stigmatization, which can be reflected in women’s choice of games where they are not visible. Comorbidity with other disorders such as depression, anxiety, bipolar disorder, personality disorders, and the use of other substances are noteworthy.

**Discussion:**

The factors explaining why female gamblers do not seek treatment compared to male gamblers are analyzed. More studies on women’s experience with gambling are needed to address the specific characteristics of gambling disorder in women.

## Introduction

Gambling disorder is categorized as a behavioral addiction characterized by recurrent and persistent gambling behavior, which produces emotional distress and leads to economic, social, and legal problems [American Psychiatric Association (APA), 2013]. Despite that gambling has traditionally been considered a male activity (Merkouris et al., 2016), studies show that these differences are decreasing because an increasing number of women are gamblers, and the starting age is becoming equal in both sexes (Lamas et al., 2018; Macía et al., 2022).

However, the profile of the female gambler is different from that of men, either due to their gambling motives or other factors such as gambling frequency and intensity (Francis et al., 2015; Kaufman et al., 2017). In fact, gambling, especially online, is increasing faster in women than in men, and women could even show more severe gambling problems (López-Gonzalez et al., 2020). This could be partly attributed to advertising campaigns targeting female audiences (Kairouz et al., 2017) and to the many gambling options currently available—which eliminates the stigma of going to physical places (McCarthy et al., 2022)—. Another factor could be the so-called telescopic phenomenon —an accelerated progression of the addiction, which is more common among women (Marks and Clark, 2017).

Regardless of their sex, people with gambling problems often find it difficult to seek help and only consider treatment as a last resort or when they have reached a critical point (Suurvali et al., 2009). However, problem gambling among women is not always recognized, and the barriers to treatment they encounter tend to be ignored, although they perceive those barriers more than men do (Holdsworth et al., 2012; Kaufman et al., 2017; Althaus et al., 2021). According to Althaus et al. (2021) among the most frequently mentioned obstacles are the desire to handle the problem personally, shame and/or stigma, the inability to admit the problem, lack of knowledge about treatment options, difficulties in going to therapy, lack of social support, lack of childcare, and issues with the treatment itself. In addition, women have more significant comorbidity with other mental disorders and tend to have a biographical history with higher rates of childhood neglect, abuse, and trauma (Li, 2007; Poole et al., 2017; Althaus et al., 2021).

Regarding gambling preferences, previous studies show that men prefer strategy games such as cards or sports betting and face-to-face games such as casino games. In contrast, women prefer non-strategic games such as bingo or slot machines (Granero et al., 2018). Concerning gambling motives, sex differences seem more evident. Men’s motivations include wanting to be in control, the playful nature of the game, sensation-seeking, and the expectation of winning large amounts of money (Estévez et al., 2017; Macía et al., 2023). By contrast, women do not gamble as much for these reasons or social reasons but as a maladaptive mechanism to escape from problems or improve their mood (Granero et al., 2018; Macía et al., 2022). Women use gambling to cope with personal issues such as loneliness, boredom, and dysphoria, so emotional distress could be a factor that maintains maladaptive gambling behavior (Ciccarelli et al., 2017; Poole et al., 2018; Estévez et al., 2023). These findings could be related to female gamblers’ increased depressive and anxious symptoms and poorer emotion regulation (Ronzitti et al., 2016). Emotion regulation is a risk factor for addictive behavior or making it harder to quit, as gambling may function as a way to escape (Weatherly and Miller, 2013). Likewise, gambling is increasingly recognized as a public health problem that requires prevention and support strategies to minimize damage in gamblers or at-risk people (Price et al., 2021). In this regard, it seems that social perception largely determines gambling-related damage. Studies such as that by López-Gonzalez et al. (2020) point out that stigma—a social construct produced by a negative perception based on stereotypes—could affect gamblers’ self-esteem and make it a barrier to early detection of the disorder and help-seeking.

Stigmatization is related to the double penalty women suffer for being women and gamblers, as these behaviors are considered masculine. Therefore, women gamblers do not conform to traditional gender roles (Martínez-Redondo and Arostegui-Santamaría, 2021). This could affect women’s self-esteem, as they face not only their addiction but also prejudices such as being branded as bad mothers, inferior workers, or evil persons who spend money that is not theirs. When gambling, they must also face sexism, verbal aggression, and micro chauvinism (Rius-Buitrago et al., 2021).

On the other hand, many studies have shown that social support is related to more successful treatment, abstinence, and lower relapse rates in gambling disorder (Tan, 2019). In contrast, lower perception of social support is linked to increased gambling-related symptoms and greater severity of gambling behavior (Yi et al., 2019; Tessier et al., 2021). In addition, studies like that of Rius-Buitrago et al. (2021) point out that female gamblers who receive treatment do so with less family support than men, and their primary support usually comes from other women (sister, mother, daughter, friend).

In the study of female with gambling disorder, it is crucial to have direct testimonies from women who suffer from gambling disorder because they provide insight into specific elements that are not easily identifiable with a quantitative approach. Consequently, we shall study the conditioning factors and particular characteristics in the development of gambling disorder in women, such as gambling motives, preferences, and/or associated pathologies that have been studied previously (Baño et al., 2021), although rarely from a qualitative perspective.

## Methods

### Participants

The sample was comprised of 18 women divided into three focus groups, aged between 30 and 68. These women were in treatment for gambling disorder, in associations belonging to the Spanish Federation of Rehabilitated Gamblers (FEJAR).

### Procedure

The result of the participation of these associations implied a non-probabilistic approach, specifically intentional sampling (Patton, 2009). The research team established the criteria to select the sample of people interviewed. These criteria were the diagnosis of a gambling disorder, being over 18 years, being a woman, and being or having been in treatment in the rehabilitation centers that were contacted. We recruited the most diverse and representative sample of women gamblers possible, so that different contexts and perspectives could be considered.

For this purpose, the associations were contacted online to send them information about the aim of the study and organize discussion groups. Before starting the study, we informed the participants their involvement was voluntary and anonymous, and we read the informed consent. At the same time, we requested their permission to record the focus groups, explaining that the recordings would be used for future transcription, but the confidentiality of the obtained data would be preserved.

The focus groups lasted about 80 min, and a semi-structured guideline was used to organize the conversation among the participating women. The pattern of the discussion group focused on the issues of understanding female gamblers’ characteristic behaviors and experiences of gambling. The sessions were audio-recorded, and the recordings were transcribed for data coding. All the testimonies were anonymized. At the beginning of each group, we attempted to identify the contextual factors of gambling that the participants considered most relevant and explanatory to understanding their current situation. Then, at a second moment, we addressed in more detail how certain elements are critical to dependence on gambling in women.

### Ethics statement

The research obtained the ethics committee’s approval from the first author’s university.

### Design and data analysis

An inductive study was designed based on the principles of grounded theory, in which the previous literature was used to make theoretical inferences about gambling disorder (Strauss and Corbin, 1998). At the same time, a “blind” inductive process was performed to extract the categories to approach a theoretical model without excessive contamination from previous theoretical frameworks. This strategy proposed by the research team was based on the need to study the emerging factors of the direct testimonies of women with gambling disorder in more detail.

The qualitative information program Atlas.Ti 22.0 was used to recode and analyze the data. The coding process was carried out by applying summarized descriptions or live codes to the transcriptions of the testimonies in the groups. To increase the reliability of the results, they were shared with different experts and some of the participants to increase the validity of our interpretations (Nag et al., 2007).

## Results

Using the grounded theory approach, the following coding phases were proposed: open, axial, and selective. They also included different coding cycles of the transcriptions of the testimonies of the women participating in the three focus groups. These three phases led to a “theoretical model” of the most outstanding factors of disorders linked to gambling. In the first phase, the predominant empirical themes in the transcripts were identified. Next, the themes were conceptualized, and finally, a theoretical model was developed showing how women’s gambling disorder have specific elements.

### Phase 1: identification of the predominant themes

Each focus group was coded in the first phase, conducting three coding cycles (Table 1).

**Table 1:** Focus group codification.

Open coding phase	Data	Number
CAB1.	Nr of quotations	621
CAB2.	Nr of codes	220
CAB3.	Nr of categories (12) and subcategories (70)	82

Substantial concepts were identified through the analytical process and the recoding of the most relevant fragments, converging into higher-order categories.

### Phase 2: conceptualization of the salient categories

**Figure 1:**
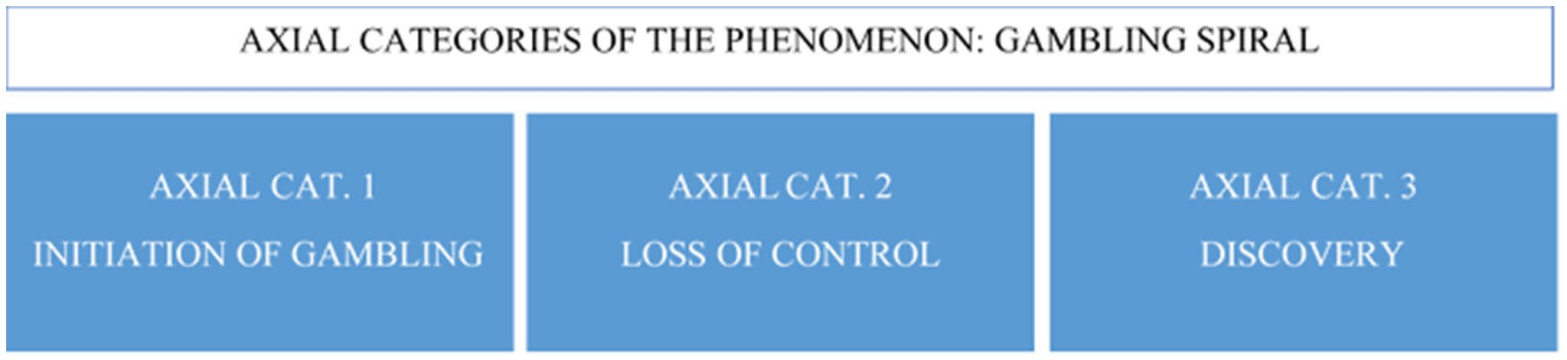
Axial categories that make up the phenomenon of gambling disorder.

In the second phase, axial coding (Strauss and Corbin, 1998) was specified to compare the emerging themes. Axial coding refines the first-order codes to higher-level categories. In this research, there were 15 axial categories. The axial coding of gambling disorder in women linked three axial categories (Figure 1), which coincide with three moments of the process in which the participants relate their experiences of dependence on gambling.

The first category comprised those moments when women say they started gambling casually and without continuity. They perceived this onset of gambling as a way to have fun, although other testimonies indicated a need for money. A more in-depth analysis of these testimonies suggested that the context of problems and stressful life events drive these women to seek a way of escape by gambling. One of the testimonies mentioned attempting to avoid loneliness and discovering how to escape from this feeling through gambling.

“I think that is when I said, I need to go ‘See, I’m at home, I feel lonely, the boys have gone for a walk, they hang out; my husband is watching football, or he does not want to go out; I’m going out by myself” 3:214 ¶ 58 Group 1.

The increase in gambling causes progressively compromising situations because the need to obtain money forces the women to seek different sources of “financing” such as loans, theft of money and jewellery from relatives and friends. This became the interviewed women’s general pattern as a way to be able to gamble. At this moment, they admit having developed a painful skill for themselves: the construction of stories helps them to conceal the situation in which they are involved. Lying becomes a constant in their lives.

“I wasn’t O.K.; I lied to everyone and said: I want one euro, give me one euro, give me two euros” 2:123 ¶ 41 in Group 2.

“I told them that I was short of money because I needed to pay for something, and I lied, I lied a lot” 2:153 ¶ 51 in Group 2.

These situations led them to become aware of the seriousness of their condition. However, they could not face the magnitude of the dynamics in which they are involved and, in some cases, manifest suicide attempts. They described two types of situations at some moment of clarity: on the one hand, the group of women who were discovered by chance and, on the other hand, those who stated that they provoked their discovery as an unconscious strategy to escape from the unbearable situation they were experiencing.

The sum of these three axial categories with their subcategories led to calling the analyzed phenomenon the gambling spiral. The participants’ experiences described a continuous and vertiginous process, with an accumulation of feelings and emotions, including anguish and pain. These emotions were difficult to control due to the different contextual variables and conditions in their lives.

Among the contextual variables in the women’s testimonies, some reported childhood abuse, including sexual abuse by parents and abuse by partners. In some cases, they were unaware of the seriousness of the impact of this maltreatment in their lives. The normalization of the attacks found in the testimonies was surprising. It was common to find in most of them a partner or a family member with alcohol problems or a consumption and/or gambling disorder that induced them to gamble.

**Figure 2:**
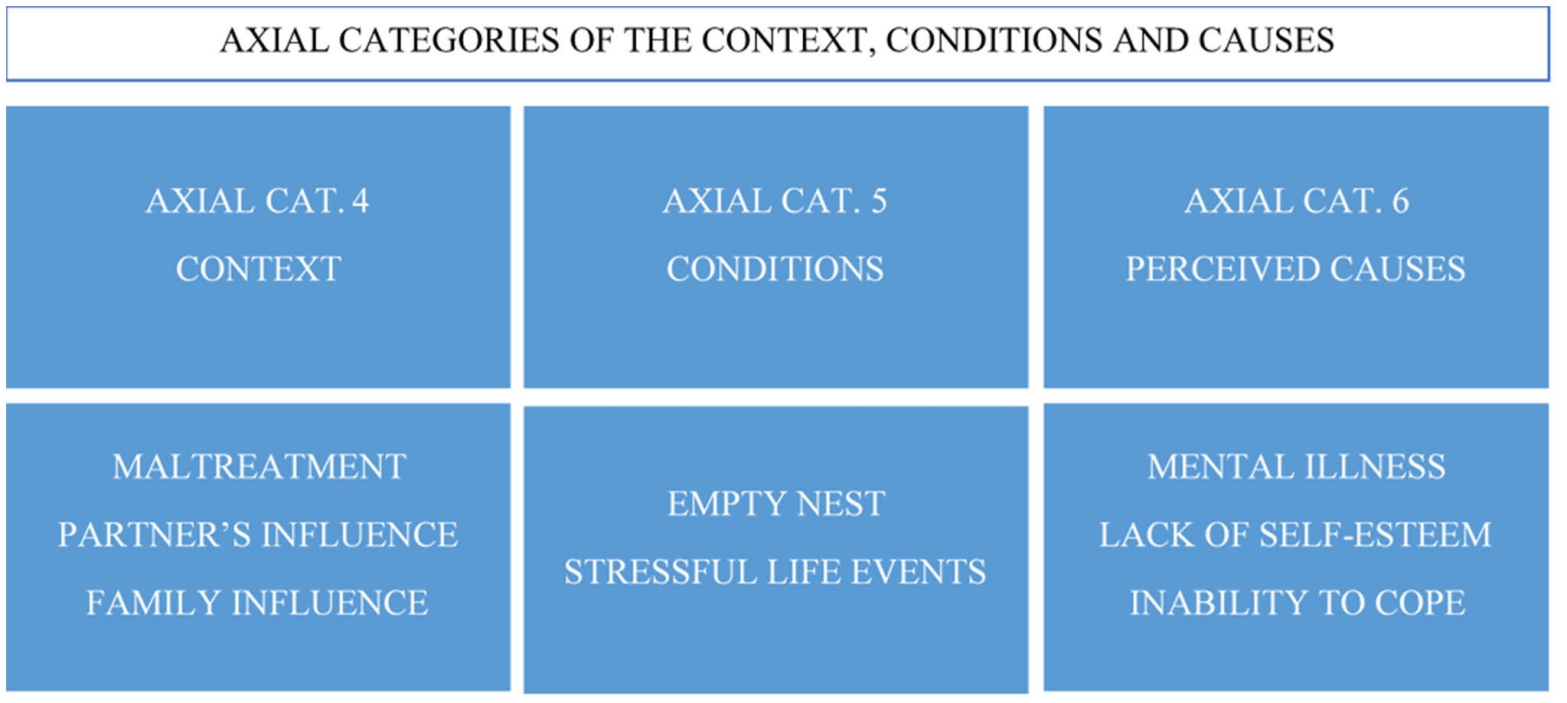
Axial categories of the context, conditions, and causes of the phenomenon.

At the same time, a series of circumstances or conditions had a catalytic effect on this spiral. The participants pointed to stressful life events such as the death of a mother, loneliness, or a child’s intellectual disability as crucial elements of their disorder. In addition, the lack of support, the inability to face problems, or the lack of self-esteem produces depression and stress. They share a feeling of overwhelming loneliness that drove them to seek an external incentive to help them to forget their stressful context and conditions, even for a few moments. This was reflected in the testimony of one of the participants (Figure 2).

“What led me to gamble? I guess loneliness, I’ve always been alone: I lied, I disappeared for three days, so many things happened to me, and later I had my son, I got pregnant and had my son” 2:141 ¶ 51 in Group 2.

**Figure 3:**
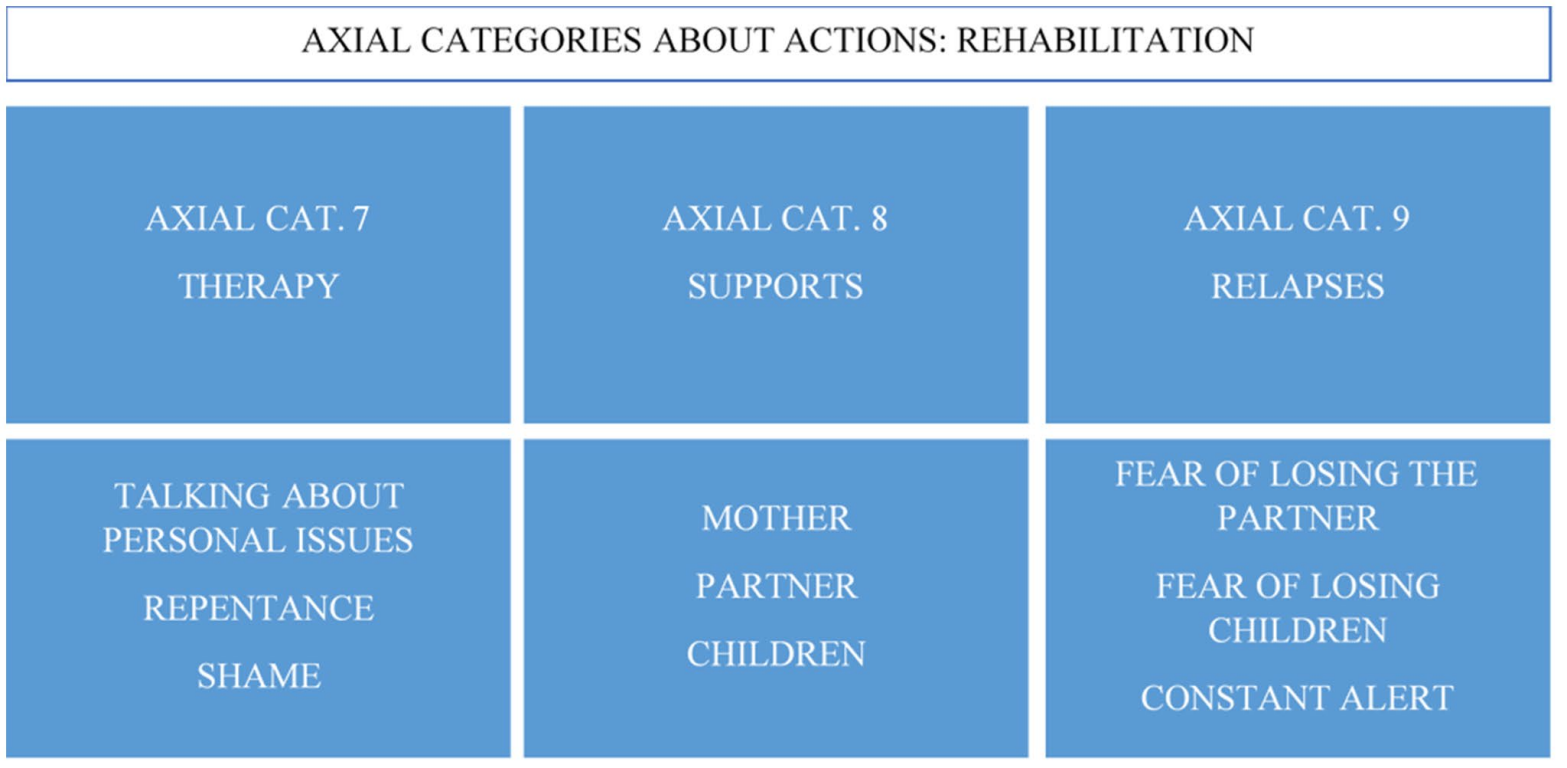
Axial categories of the actions related to gambling.

Regarding the research participants’ actions related to the gambling disorder, the analysis of results showed three new axial categories, forming the axis-category called rehabilitation (Figure 3). The therapy was considered a time where they can talk about personal things, a meeting space, and an opportunity for emotional venting. In therapy, these women could express their feelings of regret and shame, especially in the initial phases of treatment. As a result, they discovered that everything they had experienced is recognized as a disease, and this recognition helped them deal with the stigma of vice surrounding this disorder.

As these testimonies reflected:

“At the beginning (in therapy), I was ashamed because I said ‘I have not stolen to eat’; if I had stolen to eat, it’s less of a crime, I’m going to pay for it, it is still a crime, but I took money to gamble” 2:213 ¶ 65 in Group 2.

“It is embarrassing to admit that you have an illness and you need to go to therapy” 2:248 ¶ 98 in Group 2.

Concerning psychological support, the participants pointed out that support from their family environment was crucial, beginning with their mothers or their children. Their children’s support seemed essential to overcome guilt and shame and find strategies to control gambling relapses.

“They do not come to therapy because they are very ashamed. It bothers them; they say they need time for that, but the truth is that they are always there. They have not left me, nor do they feel resentment. On the contrary, now is when I need support the most” 3:370 ¶ 211 in Group 1.

“I came with my partner and my daughter, and they were shocked, they did not understand and, well, I started coming here” 4:101 ¶ 37 in Group 3.

Their children adopt the role of supervisors of the financial statements of the family unit and other indicators that can warn about a relapse. Regarding the partner’s support, their individual situations were varied. Although it was considered important, some women lost their partner’s support or had to end the relationship because it exacerbated their disorder.

“My husband came to me and told me once and no more, ‘that is nothing’, but in fact, after three months of my therapy, he said ‘goodbye, good afternoon.’ But I was not terrified that he would leave me. I was terrified that they would take my son from me for leaving him without any food. I was terrified that they would take my son away from me” 3:343 ¶ 195 in Group 1.

Relapses were another fundamental axial category in rehabilitation. It was build up through a feeling of fear, as could be seen in these words:

“If you start paying attention to your head, you go back, you relapse, and after what I’ve been through, I do not want to go through it again” 2:127 ¶ 41 in Group 2.

Whether it was the fear of losing one’s partner or losing the support of one’s children, this fear is a stimulus to be alert to possible relapses and, in some cases, a threat that weighs on them.

**Figure 4:**
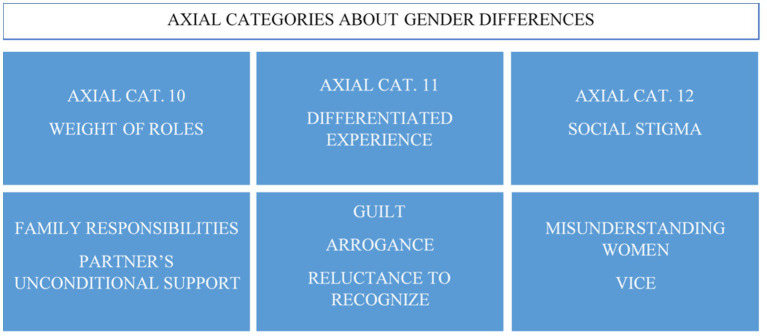
Axial categories of gender differences.

Rehabilitation may be where differences between women and men with gambling disorder are most noticeable. Co-participation in mixed therapies of both women and men made the participants directly witness the differences between the sexes. Once these axial categories were detected, work was carried out on the other axial categories, leading to the verification of the differences specified in three axial categories: the weight of gender roles, differentiated experience, and the social stigma associated with women (Figure 4).

Family responsibilities weigh differently on women and men. A cross-sectional reading of the testimonies of the axial categories of context, conditions, and support-related categories revealed differentiated elements between women and men with gambling disorder. In most cases, family responsibilities linked to the care which rests on women generated an excessive burden that these women cannot face. The issue of the empty nest was an example of this concept.

Women pointed out that they feel more guilt, in contrast to men’s reluctance to acknowledge the disorder or their display of arrogance.

“Women have more difficulties recognizing it than men. Well, actually, it may be less difficult for women to recognize it, but there is a greater difficulty for them to take the step of telling the family, both about the gambling and when they are immersed in gambling and do not want to be seen. In general, men care less” 3:182 ¶ 42 in Group 1.

Participants also pointed out that the roles assigned to women, such as house care, family responsibilities, etc., were a differentiating element in how they deal with rehabilitation because they become added difficulties.

“It is complicated because, as you said, there is a line between ‘I am responsible for money, I am responsible for the home, I am responsible for the family…’ Men do not have these responsibilities” 3:321 ¶ 147 in Group 1.

Participants even found differences in one of the key elements, the partner’s support. The participants who were also therapy partners found that the female partners were more unconditional and understanding about these men’s illness.

“In the case of men, their partner makes everything much easier for them. It may be a generalization, but it is a reality” 3:186 ¶ 46 in Group 1.

The last axial category, “social stigma,” was constructed with two elements linked to the differentiated social perception of women with gambling disorder. The participants in the study regretfully stated that they feel misunderstood as women due to their being considered negligent mothers during the spiral period of gambling. Women suffer more blame than men for this social targeting. This testimony was a clear reflection:

“We are more ashamed than men” 2:231 ¶ 79 in Group 2.

**Figure 5:**
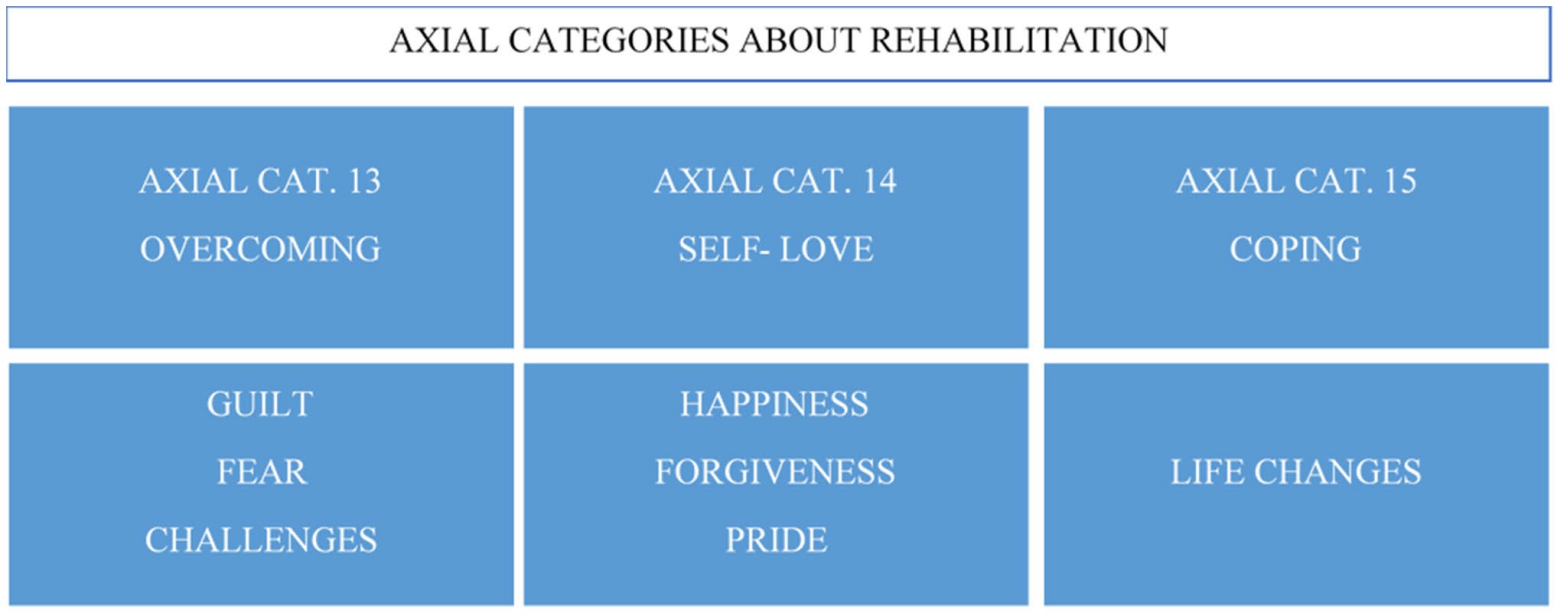
Axial categories of benefits of rehabilitation.

At the same time, this situation was aggravated by the social conception of dependence on gambling as a vice. The consideration of gambling disorder as a disease has not yet impacted society, which penalizes women more due to their female condition, a woman seen just as a mother with family responsibilities. Finally, in this phase of the axial categorization, the women participating in the rehabilitation phase described the benefits associated with the actions. These benefits are specified in Figure 5.

Participants recognized the benefits of the different strategies and actions undertaken during the rehabilitation phases. Life changes, such as decisions about work and their personal lives, had strengthened these women and provided them with the self-recognition of their improved ability to cope with their lives in general. Learning to value themselves led them to go from acknowledging the damage caused to self-forgiveness. This leads to a state of well-being and personal gratification for the road traveled and for escaping from gambling, a path not exempt from fears, guilt, and challenges, which has culminated in the feeling of succeeding.

### Phase 3: development of the theoretical model

In this last phase, the main categories were converted into aggregate dimensions. In general, first-order emerging themes became the salient categories that will form the aggregate dimensions of the theoretical framework.

Table 2 summarizes the central themes that produce the theoretical framework of the invisibility of gambling disorder in women.

**Table 2:** Main topics of the theoretical model.

Axial categories	Topics	Quotations
Axial Cat. 1 Axial Cat. 2 Axial Cat. 3	Mental disorder Persistence in the strategies to maintain gambling behavior	3:139 ¶ 3 in Group 1 It has been sort of intermittent; I have always related it to circumstances occurring in my daily life, problems, and so on that ended up going that way.	3:217 ¶ 58 in Group 1 I started by saying that I wasn’t having any fun since my son left home; I gambled, like, “Let the world know.”	2:123 ¶ 41 in Group 2 I wasn’t O.K. I lied to everyone and said: I want one euro, give me one euro, give me two euros	2:181 ¶ 65 in Group 2 I was a compulsive liar when I was playing, no? But I had to lie. I must lie; I believe that for me, lying was the normal thing to do	3:258 ¶ 75 in Group 1 We become liars, we become manipulative, and finally, we become like children.
Axial Cat. 4 Axial Cat. 5 Axial Cat. 6 Axial Cat. 10 Axial Cat. 11 Axial Cat. 12	Overload of gender roles Weight of social stigmatization	3:325 ¶ 155 in Group 1 We are bad mothers, bad wives, useless… they criticize you, even the relatives of someone who has gone through the same thing as you; they criticize you for gambling when you have to be with husband and your son. And about being a bad mother, that’s hard.
		2:172 ¶ 65 in Group 2 With the excuse that I had nowhere to go, I endured and endured, he also drank a lot, plus he also gambled.
		4:133 ¶ 47 in Group 3 In a month or so, the pandemic arrived, and I could not stand my partner any longer. Initially, my partner was very loving, but I did not realize that with all the problems and all, I was “used to” that behavior. He accommodated himself to our relationship, but in the end, I was very weak, no? I no longer had a personality or anything because he had normalized his way of being continuously on my back, not leaving me any space: “What are you doing? You have not done this right., Where are you going dressed like this? I’m ashamed to go out with you.”
		3:324 ¶ 155 in Group 1 They are sick; we are vicious
		2:116 ¶ 37 in Group 2 He had a headache, and I used to ignore it. I was in my own world; it was only me and gambling; I would often say: “fuck! If I love my son more than my life, how can I become a bad mum?” because, later, I considered myself bad because I was bad.
		3:321 ¶ 147 in Group 1 It is complicated because there is, as you said, a line between “I have to be responsible for money, I have to be responsible for the house, I have to be responsible for the family…” men do not have this responsibility.
		2:206 ¶ 65 in Group 2 I thought I was vicious; it took a lot of effort to recognize that it was an illness
Axial Cat. 7 Axial Cat. 8 Axial Cat. 9	Professional support Family support	3:362 ¶ 209 in Group 1 They directly cut off contact, they did not want to spend Christmas with us, and that’s very hard, and it hurts a lot. You always say, “and why did I do this and why…” and you relate everything that happened to “if you had not done some things, it would not have been your fault, and would not have happened.” 3:347 ¶ 203 in Group 1 My son is the one who helped me. In fact, in his room, on his board, he has a photo of him and me.	4:102 ¶ 37 in Group 3 Going to a psychologist is always complicated, and if you do not connect well… I connected very well with her, and she with me, and then I decided that I needed more than one session per month, so I talked with her, and she said yes, she would attend to me, but I needed to tell this in the association. So I informed them in the association, and they said there was no problem with that, so since then, I’ve been going once a week.
Axial Cat. 13 Axial Cat. 14 Axial Cat. 15	Resilience	2:114 ¶ 37 in Group 2 I have been here 14 months, but until I was here for 9 months, I did not realize that I must love myself: love myself so I can love others.
		4:83 ¶ 27 in Group 3 I’m very proud of myself, very proud to have gotten out, so I do not care what others think	3:201 ¶ 56 in Group 1 Any problem you have at home, but now, I’m happy with myself. In fact, I found a permanent job. I got my driving license when it was unthinkable that I could have achieved it because it was something... it was taking a steering wheel, and I got my driving license.	4:157 ¶ 59 in Group 3 I’m studying, and I passed two courses in a year. And I have gotten excellent grades. I have seen my classmates and could laugh with them, although I still cannot appreciate it sometimes. When I think that I have done it… I do not recognize it yet, but I hope to go forward…

## Discussion

This study has aimed to analyze women’s gambling experience. The results lead to a theoretical model that addresses the problem of gambling disorder in women based on two axes: a first axis that indicates the excessive burden of gender roles in the gambling spiral, which remains in the rehabilitation and therapy phases; and a second axis, in which professional and family support are combined, becoming the resilient strategy to address women’s dependence on gambling. The results have shown that women may start gambling due to childhood ill-treatment and/or abuse (generally perpetrated by parents or very close figures), partner abuse, or a family history of relatives who have problems with alcohol, substance consumption, or gambling disorder. In this latter case, it is often the family members themselves who initiate the women into gambling. Likewise, the testimonies show that the initiation of gambling could be due to difficulty coping with stressful life events (e.g., the death of one of the parents or relatives, loneliness, or a child’s illness). This difficulty can lead to a lack of self-esteem, depression, and/or stress. In this sense, gambling could become a tool to deal with loneliness and manage emotions. The study participants’ accounts reinforce previous studies of female gamblers, which state that gambling becomes a maladaptive way of managing negative emotions or childhood experiences of neglect, trauma and/or abuse (Poole et al., 2017, 2018; Lelonek-Kuleta, 2022; Estévez et al., 2023).

Regarding social stigmatization, stories about feeling misunderstood predominate. It has been observed that female gamblers are branded as negligent, vicious, and bad mothers during the gambling spiral, generating feelings of guilt. Traditionally, family caregiving responsibilities have disproportionally fallen on women, which is still prevalent nowadays (Lamas et al., 2018). However, their status as “female gamblers” seems to distance them from the role of caregivers and mothers from the social perspective, generating feelings of rejection even in their family environment (spouses and/or children). Previous studies show that stigmas, prejudices, and gender roles could lead to self-messages and feelings of guilt and shame, affecting mental and somatic health, as well as help-seeking and rehabilitation (Dunn et al., 2012; Lamas et al., 2018; Rius-Buitrago et al., 2021).

Another essential aspect is the small number of women who attend gambling rehabilitation resources. Macía and Estévez (2022) point out that socially, women with gambling problems continue to be considered “vicious” and not sick people. This could help to explain why only a few women obtain and persist in treatment. These observations provide hints about how to adjust treatment for women with gambling disorder. Often, female gamblers do not have enough family or social support to rehabilitate and may even suffer contempt or abuse by their partners (Lamas et al., 2018). Conversely, men seem to receive more family support and social justification (Echeburúa et al., 2014; Martínez-Redondo and Luján-Acevedo, 2020). This aspect is critical to rehabilitation. In their accounts, the women highlight that their children’s support is vital to overcome guilt and shame and that family accompaniment is a protective factor against relapses. It would be interesting to develop this line of research further and implement practices that promote increased support from the immediate environment.

On the other hand, women with gambling disorder frequently present comorbidity with psychiatric disorders such as depression, anxiety, bipolar disorder, personality disorders, and the use of other substances (Desai and Potenza, 2008; Ronzitti et al., 2016; Macía et al., 2023). Comorbidity is highly related to the severity of gambling problems and their consequences, which include suicide, the leading cause of death in female gamblers (Althaus et al., 2021).

The data from this study raise the possibility that suicidal thoughts emerge as a consequence of debts, depression, lack of support, loneliness, and coping difficulties. However, recent studies indicate that psychiatric comorbidity does not entirely explain the relationship between gambling and suicidal ideation, suggesting other underlying mechanisms, such as financial stress, feelings of loneliness, the impact on family life, or relational arguments (Wardle et al., 2020). Therefore, it would be interesting to consider comorbidity with other disorders and the typical experiences of gambling addiction as risk indicators for suicidal ideation and behavior so that suicide prevention protocols can be implemented.

Finally, women seem to prefer games that consume time but do not have the aim of socializing, such as scratch cards, bingo, or slot machines, data that agree with those obtained by Marcos and Choliz (2019) and by Li (2007). In this sense, even though loneliness is a risk factor for women with gambling disorder, testimonies suggest that women prefer games that allow them to gamble without being seen, in order to avoid social stigma. They mostly avoid going to places like betting houses because they feel judged, sometimes receiving disparaging comments from others, even other gamblers. However, despite the different consequences and the social perception of women with gambling disorder, there is still a lack of research and resources incorporating the gender perspective.

## Limitations

Despite the study’s contributions, it is not exempt from limitations. This study is a first approach to the specificities of gambling disorder in women from a gender perspective, but future studies should contrast these findings with a group of men. This would serve to objectify the perceived gender differences in gambling disorder. Another limitation of the study is the sample. Women approach resources less than men, so finding a group of women is difficult (Braun et al., 2014; Lamas et al., 2018). However, evidence indicates that women attend primary care resources more frequently than men (Markez et al., 2004), which could explain the lack of women in gambling rehabilitation centers. On the other hand, despite being part of the same association, the women who made up the groups in this study frequently did not know each other, which could have affected them when they shared their experiences.

## Conclusion

To date, there has been limited research in women’s gambling experience. However, factors such as prejudice (especially against women who violate social norms) and support-seeking seem crucial to understanding the distinctive phenomenon of gambling in women and conducting gender-specific treatments and preventions. In addition, a better understanding of the mechanisms and stigmas underlying gambling disorder in women may serve as prevention for risky behaviors, such as suicidal ideation.

## Data availability statement

The datasets presented in this article are not readily available because data is not available due to confidentially reasons. Requests to access the datasets should be directed to lauramacia@deusto.es.

## Ethics statement

The Institutional Review Board of the first author’s university approved the study (ETK-17/20-21). This study was performed following the principles of the Declaration of Helsinki. Informed consent was obtained from all the participants included in the study. Written and oral informed consent was obtained from the individuals for the publication of any potentially identifiable data included in this article.

## Author contributions

AE: Project administration, Resources, Supervision, Writing – review & editing. LM: Conceptualization, Funding acquisition, Methodology, Supervision, Writing – review & editing. AO: Investigation, Writing – original draft. MA: Formal Analysis, Writing – original draft.

## References

[ref1] Althaus J. Zendle D. Bowden-Jones H. (2021). “Gambling and gaming addictions in women” in Textbook of addiction treatment. eds. Carrà G. Galanter M. Baldacchino A. M. (Cham: Springer)

[ref2] American Psychiatric Association (APA) (2013). Diagnostic and statistical manual of mental disorders-text revision. 5th Edn. Arlington, VA: American Psychiatric Association.

[ref3] Baño M. Mestre-Bach G. Granero R. Fernández-Aranda F. Gómez-Peña M. Moragas L.. (2021). Women and gambling disorder: assessing dropouts and relapses in cognitive behavioral group therapy. Addict. Behav. 123:107085. doi: 10.1016/j.addbeh.2021.107085, PMID: 34425460

[ref4] Braun B. Ludwig M. Sleczka P. Bühringer G. Kraus L. (2014). Gamblers seeking treatment: who does and who doesn’t? J. Behav. Addict. 3, 189–198. doi: 10.1556/JBA.3.2014.3.7, PMID: 25317343 PMC4189314

[ref5] Ciccarelli M. Griffiths M. D. Nigro G. Cosenza M. (2017). Decision making, cognitive distortions and emotional distress: a comparison between pathological gamblers and healthy controls. J. Behav. Ther. Exp. Psychiatry 54, 204–210. doi: 10.1016/j.jbtep.2016.08.012, PMID: 27592413

[ref6] Desai R. A. Potenza M. N. (2008). Gender differences in the associations between past-year gambling problems and psychiatric disorders. Soc. Psychiatry Psychiatr. Epidemiol. 43, 173–183. doi: 10.1007/s00127-007-0283-z 18080792 PMC3700360

[ref7] Dunn K. Delfabbro P. Harvey P. (2012). A preliminary, qualitative exploration of the influences associated with drop-out from cognitive-behavioural therapy for problem gambling: an Australian perspective. J. Gambl. Stud. 28, 253–272. doi: 10.1007/s10899-011-9257-x, PMID: 21643763

[ref8] Echeburúa E. Salaberría K. Cruz-Sáez M. (2014). Nuevos retos en el tratamiento del juego patológico. Ter. Psicol. 32, 31–40. doi: 10.4067/S0718-48082014000100003

[ref9] Estévez A. Jáuregui P. Sánchez-Marcos I. López-González H. Griffiths M. D. (2017). Attachment and emotion regulation in substance addictions and behavioral addictions. J. Behav. Addict. 6, 534–544. doi: 10.1556/2006.6.2017.086, PMID: 29280395 PMC6034944

[ref10] Estévez A. Macía L. Macía P. (2023). Looking at sex differences in gambling disorder: the predictive role of the early abandonment schema, gambling motives and alexithymia in depression. J. Gambl. Stud. 39, 1815–1832. doi: 10.1007/s10899-023-10251-w, PMID: 37733147 PMC10628046

[ref11] Francis K. L. Dowling N. A. Jackson A. C. Christensen D. R. Wardle H. (2015). Gambling motives: application of the reasons for gambling questionnaire in an Australian population survey. J. Gambl. Stud. 31, 807–823. doi: 10.1007/s10899-014-9458-1 24705633

[ref12] Granero R. Fernández-Aranda F. Mestre-Bach G. Steward T. García-Caro B. Prever F.. (2018). Clustering of treatment-seeking women with gambling disorder. J. Behav. Addict. 7, 770–780. doi: 10.1556/2006.7.2018.93, PMID: 30238785 PMC6426395

[ref13] Holdsworth L. Hing N. Breen H. (2012). Exploring women's problem gambling: a review of the literature. Int. Gambl. Stud. 12, 199–213. doi: 10.1080/14459795.2012.656317

[ref14] Kairouz S. Monson E. Robillard C. (2017). “Gender comparative analysis of gambling patterns in Canada” in Gambling disorders in women: An international perspective on treatment and research. eds. Bowden-Jones H. Prever F. (London: Routledge).

[ref15] Kaufman A. Jones Nielsen J. D. Bowden-Jones H. (2017). Barriers to treatment for female problem gamblers: a UK perspective. J. Gambl. Stud. 33, 975–991. doi: 10.1007/s10899-016-9663-1, PMID: 28008550 PMC5579153

[ref16] Lamas J. L. Santolaria R. Estévez A. Jauregui P. (2018). Guía clínica específica “Mujer y juego.” [Specific clinical guideline “Women and gambling”]. Delegación del Plan Nacional Sobre Drogas.

[ref17] Lelonek-Kuleta B. (2022). Gambling motivation model for older women addicted and not addicted to gambling–a qualitative study. Aging Ment. Health 26, 639–649. doi: 10.1080/13607863.2021.1895068 33724116

[ref18] Li J. (2007). Women's ways of gambling and gender-specific research. Sociol. Inq. 77, 626–636. doi: 10.1111/j.1475-682X.2007.00211.x

[ref19] López-Gonzalez H. Russell A. M. Hing N. Estévez A. Griffiths M. D. (2020). A cross-cultural study of weekly sports bettors in Australia and Spain. J. Gambl. Stud. 36, 937–955. doi: 10.1007/s10899-019-09898-1, PMID: 31606863

[ref20] Macía L. Estévez A. (2022). La ludopatía no es sólo cosa de hombres [compulsive gambling is not just a male thing]. The conversation (ISSN 2201-5639). Available at: https: //theconversation.com/la-ludopatia-no-es-solo-cosa-de-hombres-187098

[ref21] Macía L. Jauregui P. Estévez A. (2022). Gambling: exploring the role of gambling motives, attachment and addictive behaviours among adolescents and young women. J. Gambl. Stud. 39, 183–201. doi: 10.1007/s10899-022-10124-8, PMID: 35579778 PMC9981506

[ref22] Macía L. Jauregui P. Herrero M. Iruarrizaga I. Micó V. Lamas J.. (2023). Sex-comparative study of gambling disorder regarding alexithymia and symptoms of depression, anxiety and hostility. Compr. Psychiatry 122:152364. doi: 10.1016/j.comppsych.2023.152364 36682199

[ref23] Marcos M. Choliz M. (2019). Mujer y juego online. Propuesta de tratamiento de un caso de adicción a videobingo [Women and online gambling. Treatment proposal for a case of addiction to videobingo]. Información Psicol. 117, 99–114. doi: 10.14635/IPSIC.2019.117.7

[ref24] Markez I. Póo M. Romo N. Meneses C. Gil E. Vega A. (2004). Mujeres y psicofármacos: la investigación en atención primaria [Women and psychotropic drugs: research in primary care]. Rev. Asociac. Española Neuropsiquiatría 91, 37–61.

[ref25] Marks K. R. Clark C. D. (2017). The telescoping phenomenon: origins in gender bias and implications for contemporary scientific inquiry. Subst. Use Misuse 53, 901–909. doi: 10.1080/10826084.2017.1385079, PMID: 29161174 PMC6129392

[ref26] Martínez-Redondo P. Arostegui-Santamaría E. (2021). Situación en España de la Violencia de Género y el abuso de sustancias [The situation of gender violence and substance abuse in Spain] Madrid: Ministerio de Sanidad

[ref27] Martínez-Redondo P. Luján-Acevedo F. (2020). Hombres y adicciones. Intervención desde perspectiva de género [Men and addictions. Intervention from a gender perspective]. Madrid: UNAD.

[ref28] McCarthy S. Thomas S. Marko S. Pitt H. Randle M. Cowlishaw S. (2022). Women’s perceptions of strategies to address the normalization of gambling and gambling-related harm. Aust. N. Z. J. Public Health 46, 821–828. doi: 10.1111/1753-6405.13264, PMID: 35735793

[ref29] Merkouris S. S. Thomas A. C. Shandley K. A. Rodda S. N. Oldenhof E. Dowling N. A. (2016). An update on gender differences in the characteristics associated with problem gambling: a systematic review. Curr. Addict. Rep. 3, 254–267. doi: 10.1007/s40429-016-0106-y

[ref30] Nag R. Corley K. G. Gioia D. A. (2007). The intersection of organizational identity, knowledge, and practice: attempting strategic change via knowledge grafting. Acad. Manag. J. 50, 821–847. doi: 10.5465/amj.2007.26279173

[ref31] Patton M. Q. (2009). Qualitative research and evaluation methods. 3rd Thousand Oaks. CA: Sage.

[ref32] Poole J. C. Dobson K. S. Pusch D. (2018). Do adverse childhood experiences predict adult interpersonal difficulties? The role of emotion dysregulation. Child Abuse Negl. 80, 123–133. doi: 10.1016/j.chiabu.2018.03.006, PMID: 29604503

[ref33] Poole J. C. Kim H. S. Dobson K. S. Hodgins D. C. (2017). Adverse childhood experiences and disordered gambling: assessing the mediating role of emotion dysregulation. J. Gambl. Stud. 33, 1187–1200. doi: 10.1007/s10899-017-9680-8 28258336

[ref34] Price A. Hilbrecht M. Billi R. (2021). Charting a path towards a public health approach for gambling harm prevention. J. Public Health 29, 37–53. doi: 10.1007/s10389-020-01437-2, PMID: 33432287 PMC7787930

[ref35] Rius-Buitrago A. Soriano-Villarroel I. López-González H. (2021). “Yo sabía que nadie me iba a juzgar”: La adicción al juego online desde la perspectiva de género [“I knew no one would judge me”: Online gambling addiction from a gender perspective]. Musas 6, 138–155. doi: 10.1344/musas2021.vol6.num1.8

[ref36] Ronzitti S. Lutri V. Smith N. Clerici M. Bowden-Jones H. (2016). Gender differences in treatment-seeking British pathological gamblers. J. Behav. Addict. 5, 231–238. doi: 10.1556/2006.5.2016.032, PMID: 27348561 PMC5387774

[ref37] Strauss A. L. Corbin J. M. (1998). Basics of qualitative research: Techniques and procedures for developing grounded theory (2nd). Thousand Oaks, CA: Sage.

[ref38] Suurvali H. Cordingley J. Hodgins D. Cunningham J. (2009). Barriers to seeking help for gambling problems: a review of the empirical literature. J. Gambl. Stud. 25, 407–424. doi: 10.1007/s10899-009-9129-9, PMID: 19551495

[ref39] Tan K. A. (2019). The effects of personal susceptibility and social support on internet addiction: an application of Adler’s theory of individual psychology. Int. J. Ment. Heal. Addict. 17, 806–816. doi: 10.1007/s11469-018-9871-2

[ref40] Tessier S. Romo L. Zerhouni O. (2021). Impact of advertising campaigns among online gamblers: the role perceptions of social support and personality traits. Front. Psych. 12:599988. doi: 10.3389/fpsyt.2021.599988, PMID: 34764890 PMC8576387

[ref41] Wardle H. John A. Dymond S. McManus S. (2020). Problem gambling and suicidality in England: secondary analysis of a representative cross-sectional survey. Public Health 184, 11–16. doi: 10.1016/j.puhe.2020.03.024, PMID: 32409100

[ref42] Weatherly J. N. Miller K. B. (2013). Exploring the factors related to endorsing gambling as an escape. Int. Gambl. Stud. 13, 52–64. doi: 10.1080/14459795.2012.703214

[ref43] Yi G. Huang L. Lam A. I. Latkin C. Hall B. J. (2019). Spatial and sociodemographic correlates of gambling participation and disorder among female Filipino migrant workers in Macao, People’s Republic of China. Addict. Behav. 97, 49–55. doi: 10.1016/j.addbeh.2019.05.021 31146151

